# Modeling immune response and its effect on infectious disease outbreak dynamics

**DOI:** 10.1186/s12976-016-0033-6

**Published:** 2016-03-05

**Authors:** Jorge Reyes-Silveyra, Armin R. Mikler

**Affiliations:** Center for Computational Epidemiology and Response Analysis, University of North Texas, 1155 Union Circle 311277, Denton, 76203 TX USA; Department of Computer Science and Computer Engineering, Pacific Lutheran University, 1010 S 122nd St, Tacoma, 98447 WA USA

**Keywords:** Mathematical and computational modeling, Immunology, Epidemiology, Socio-demographics, Heterogeneous populations

## Abstract

**Background:**

In recent epidemiological models, immunity is incorporated as a simplified value that determines the capacity of an individual to become infected or to transmit the disease. Moreover, the quality of the immune response determines the chances of infection and the length of time an individual is capable to infect others. We present a model that incorporates individuals’ immune responses to, further, examine the role of the collective immune response of individuals in a population during an infectious outbreak.

**Methods:**

We constructed a contagion model that incorporates the collective immune response of individuals represented by the superposition of individual immune responses (PIR). Multiple probability distributions are used to represent the immunocompetence of different age groups, thereby modeling the concept of Population Immune Response (PIR). Multiple experiments were conducted in which the population is divided in different age groups for which each group has a unique immune response quality and thus a different length for its immune periods. Finally, we explored the effects of implementing different vaccination strategies in the population.

**Results:**

The experiments displayed important variations in the outbreak dynamics as a consequence of incorporating PIR in homogeneous and mixed populations. The experiments showed that individuals with weak immune responses and those who are immune to the pathogen play a significant role in shaping the outbreak dynamics. Finally, after implementing different vaccination strategies, the results suggest that if vaccination resources are limited, the vaccination should be targeted towards individuals that spread the disease for a longer period of time.

**Conclusions:**

Our results suggest that it is essential for the public health establishment to increase their understanding of the characteristics of regional demographics that could impact the quality of the immune response of the individuals. The results indicate that it is necessary to further investigate mitigation strategies to limit the capacity to transmit the disease by individuals that spread the pathogen for extended periods of time. Ultimately, this study suggests that it is crucial for public health researchers to identify appropriate targeted vaccination regimes and to explore the link between PIR and outbreak dynamics to improve the monitoring and mitigating efforts of ongoing and future epidemics.

## Introduction

The immune system of an organism provides an extraordinary defense against foreign attacks. Once it recognizes matter as non-self, it activates multiple chemical and physiological processes to control and eliminate the pathogen [1]. These processes are collectively known as immune response. The immune system mounts a response in an attempt to stop the growth of an invading organism in order to retain optimal functionality of the host. Controlling such proliferation is beneficial for the organism, since the quantity of foreign material affects the amount of time during which the organism experiences infection. Further, the duration of the infectious period, during which an organism might transmit the infection to others, is directly related to the quantity of foreign material in the host. Hence, we conjecture that the dynamics of an infectious disease epidemic in a population are driven, among other things, by the organisms’ immune responses. This research establishes this relationship through the integration of immune response into the population at large.

The most recent approaches to infectious disease outbreak modeling incorporate non-homogeneous components to the individuals to be modeled. Studies of the effects of non-homogeneous populations on the dynamics of infectious outbreaks have shown the importance of integrating individuals with heterogeneous characteristics [2]. Although the amount of time a person is capable to transmit the disease varies among individuals, many models set that value to be homogeneous for the population. Season and temperature are some of the sources that influence that difference [3, 4]. However, we highlight the capacity of the immune response to diminish the pathogen as a crucial component for that variation as well.

In this paper, we describe the types of immune responses that occur during an infection, as well as conditions that can contribute to variations in immunocompetence among individuals. Further, we discuss the relationship between immune response efficacy and the length of the individuals’ infectious and latent periods. Next, we present two methods to calculate the quality of the immune response of an individual. Most importantly, we introduce the concept of Population Immune Response (PIR). This new concept defines the collective immune response of individuals in a population as the superposition of individual immune responses.

## Background

### Immune response and infection

To understand the role of the immune response during an infectious process, it is necessary to address some essential concepts related to it. The primary elements of a human immune response and the factors that determine their quality are defined below. Further, the progression of an infectious disease in an individual after exposure to a pathogen and the antagonistic role of the immune response are examined. Finally, the concept of viral/bacterial load and its relation to the length of the infection periods is described.

#### Immunity

The survival of an organism is highly correlated with the quality of its immune system [5, 6]. The immune system provides two types of defense against invaders: *innate* and *adaptive* immunity. The innate response is non-specific to the pathogen and does not provide a long lasting immunity. The major components of the innate response are:

- *Physical barriers*, such as tears and skin, block the entrance of possible invaders.
- The *Complement system* is composed of molecules that intensify the effect of other immune functions.
- *Macrophages* are responsible to phagocytose, digest and present pathogens.
- *Natural Killer Cells* induce cytotoxic apoptosis (cell death) to infected cells.

A pathogenic invasion occurs once viral or bacterial material pass these first lines of defense. Once they have crossed the innate defense, pathogens tend to migrate to suitable locations for occupation and multiplication. Foreign invasion activates an adaptive immune response that impedes the replication and migration of the pathogen to attempt to free the host from the external threat. The adaptive response is specific to each invader and is conducted by two main types of cells:

- *T-Cells* : Lymphocytes maturated in the thymus.
- *B-Cells* : Lymphocytes maturated in the bone marrow.

T-cells are highly specialized cells that not only coordinate (*T-helper*) and regulate (*T-regulatory*) the immune response, but also destroy infected cells (*T-cytotoxic*). B-cells secrete antibodies and perform pathogen presentation similar to the macrophages. Antibodies are proteins that can mark an infected cell or a pathogen to facilitate its elimination. Additionally, antibodies can stop the replication of the pathogens by impeding their attachment to healthy cells. Both T-cells and B-cells provide immunity against a pathogen by producing memory cells (T-memory and B-memory) during an infection process. Similarly, immunity can be artificially induced by vaccination. Immunity against a pathogen heightens the immune response to prevent future infections.

The efficacy of the immune response is determined by multiple factors. Many of them are associated with the host, including age, physical fitness, gender, and nutrition [7]. Various studies report different causes for deterioration of the immune system throughout an individual’s life-time. As the individual ages, limited capacity to defend against invaders is caused by multiple alterations in T-cell and B-cell functionality [8, 9]. Likewise, nutrition is a critical factor for the quality of the immune response of an individual. Studies have shown a strong relationship between malnutrition and multiple immune response deficiencies. These may include impairment of the complement system, cell mediated immunity, and phagocyte functionality [10]. Obesity [11], high-cholesterol levels [12], and low vitamin and mineral intake [13] are some of the nutrition related causes for those immune response deficiencies.

In addition to access to health care, types and frequency of social interactions, gender drastically affects the competence of the immune response [14, 15]. This is primarily attributed to the blood levels of gonadal steroid hormones. Multiple studies have shown androgens as natural immunosuppressors, as well as estrogens as humoral immunity enhancers [16]. Moreover, physical fitness of individuals has a unique effect on the immune response. Although positive immuno-stimulatory activity is observed with moderate exercising, both lack of and excessive exercise produce an immuno-suppressive response [17, 18].

### Infection

Diseases in individuals develop in sequential phases [19]. It is known that once a pathogen invades a susceptible individual, he or she progresses through different infection stages: Latent, Infectious and Recovered/Removed. The progression from one stage to another occurs after the consummation of different time periods. These periods are known as *latent period* and *infectious period*. The latent period is the amount of time necessary for an individual to develop the capacity to infect others. Analogously, the infectious period is the amount of time during which an individual is capable of transmitting the disease to others.

An infectious agent replicates after it penetrates the host’s basic defenses. The efficacy of the replication process can be quantified through the corresponding *viral/bacterial load* (*vbl*) [20]. The *vbl* is the concentration of virus or bacteria in plasma at a certain moment in time [1]. The *vbl* value is commonly used as an indicator for disease severity [21] and the host’s capacity of transmitting it [22]. Since the immune system is responsible for controlling *vbl* in the host, we conceive a direct relationship between them. A stronger immune response restrains the growth of the external threat more efficaciously; Hence, we conclude that the quality of the immune response affects the quantity of *vbl* during infection.

We define 4 types of immune responses based on their quality: . The quality of each response is determined by the severity of the infection and the length of the infectious period of an individual with that type of response. A standard immune response  represents the average response in a healthy individual. An individual with this response remains infected for the same amount of time as the majority of the population. Individuals with this response usually recover after the infection is eradicated and rarely succumb to the disease. Individuals with the hyperimmune response  will stay infected for a shorter period of time than the majority of the population.  represent the immune response of those individuals with a superior count of pathogen specific immune cells as compared to individuals with an average immune response. This type of response guarantees the survival of the individual throughout infection. The hypoimmune response  represents the immune response of individuals with an immunocompromised immune system (eg. Cancer, Diabetes or HIV subjects). A person with this response is infectious for a longer period of time than the majority of the population. Individuals with this response have increased probabilities to succumb to the disease.  comprises individuals that are actively or passively immune against a specific pathogen. Elements of the population with this type of response will get infected for a short period of time, but never become symptomatic or infectious.

**Fig. 1:**
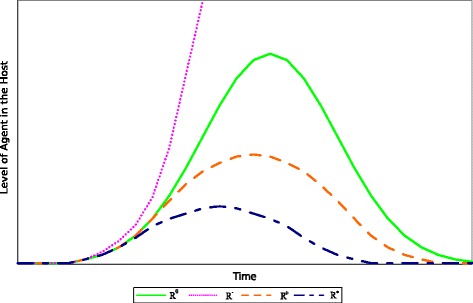
Four contrasting scenarios that depict the viral load in an individual over time as a function of the quality of their immune response

To illustrate these concepts and their relationship to *vbl*, Fig. 1 portrays four contrasting scenarios of a primary viral infection.  results in consistent pathogen replication until the immune response is strong enough to overcome the infection and eliminate it.  produces a similar effect, but it is more effective than  In contrast,  is not capable of containing the infection. This will result in uncontrolled growth of the virus leading to chronic infection or death of the host. On the opposite side of the spectrum,  is more efficient at limiting pathogen replication than all other responses. This results in smaller quantity of *vbl* in the host at all times.

**Fig. 2:**
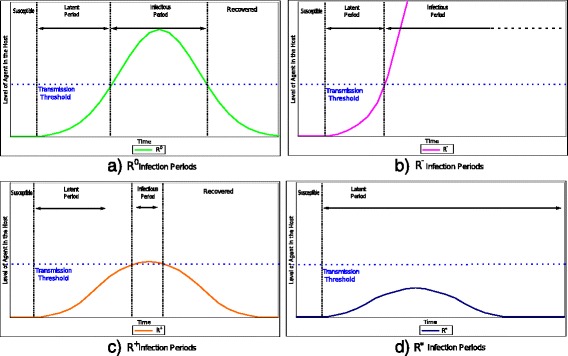
Duration of the infectious periods for each scenario presented in Fig. 1 as a function of *vbl*

The integration of *vbl* can be utilized as a threshold to determine the commencement and termination of the disease periods during an infection [23]. Figure 2 depicts the duration of the time periods for each scenario presented in Fig. 1. The length of each period is determined by a transmission threshold. The transmission threshold (*vbl*^∗^) is established as the quantity of *vbl* that is necessary for an individual to become infectious. The latent stage commences at the infection point and terminates once *vbl* surpasses *vbl*^∗^. Equivalently, the length of the infectious period starts at the end of the latent period and culminates once the *vbl* falls below *vbl*^∗^. For example, Fig. 2b portrays a hypoimmune response  during an infectious process. In this figure, the *vbl* growth rate at the beginning of infection is similar to the rest of the other responses. Once *vbl* exceeds *vbl*^∗^, the immune response is not capable of containing the pathogen proliferation. An individual with  may experience a long or a short infectious period depending on its capacity to fight the disease. If infection becomes chronic, the individual is infectious period will be longer than that of an individual with the standard immune response  Otherwise, if the individual succumbs to the infection, it will be moved to the Recovered/Removed population earlier than the average individuals, resulting in a short infectious period. An individual with a hyperimmune response  will have a shorter than average infectious period. Figure 2c represents such a scenario. We observe that *vbl* in Fig. 2c exceeds the threshold for a brief amount of time, resulting in a short infectious period. Finally, since the  response does not exceeds *vbl*^∗^, it results in a long latent period and the individual never progresses to infection. This scenario is depicted in Fig. 2d.

In general, these scenarios illustrate how the length of the disease periods are a function of the viral load. It is possible to obtain those values from sources such as immunological, mathematical, or computational models. The model selected must simulate the interaction between the pathogen and the immune system. Ultimately, from the model, *vbl* is obtained to determine the length of the disease periods for each individual. In the Methods section below, we present an example to determine the infectious periods by simulating the disease trajectory in a host with the use of a mathematical model.

### Immune response and epidemiology

Epidemiologists have made use of different approaches to incorporate immunological factors into their models. Some models implement immunity as an effect of a vaccination strategy [24] or as a period in which the individual cannot be infected after a primary infection [25]. Moreover, other scientists have engaged in a comprehensive study of the relationship between immune response and the population. Hellriegel [26] presents a review of that relationship. In this review, the importance of integrating immunity and epidemiology (immunoepidemiology) is highlighted. The author defines three different approaches for this integration: Within-host, between-host and individual-to-population dynamics. Furthermore, the author proposes different combinations of those approaches to assess the role of immunity in determining epidemiological patterns. Following, we present a description of multiple epidemiological models that incorporate immunologic factors.

Dushoff [27] depicted a model in which the probability of the disease progressing in an infected individual is not only determined by its own characteristics, but by the level of disease in the population. That assumption is based on the suggestion that an exposure with low pathogen load may lead to immunity or short lasting infection and minimum disease transmission, while an exposure with high pathogen load may lead to a longer infection and to greater transmission of the disease. To implement that assumption, the model contains two individual classes: heavily infected and lightly infected. Individuals can depart from one class to another as a function of the force of infection.

Martcheva and Pilyugin [28] presented a Susceptible-Infectious-Recovered model in which the immune status of the individuals increases during the infectious period. In this model, the initial absence of immunity of individuals sets all of them in the susceptible group. However, once an individual becomes infected its immune status increases over the course of infection. After an individual recovers from the disease, a possible reinfection is restricted by its immune status. However, immunity may decrease as a function of time, increasing the probability of a secondary infection.

Vickers and Osgood [29] introduced a mathematical framework of population infection dynamics in which individuals mount an immune response in response to infection and the contacts between them are distributed in a simple contact network. The immune response is represented by a population of differentiated and non-differentiated cytotoxic immune cells. The social network is implemented by placing each individual in a Poisson distributed network such that the incoming viral load of an individual is proportional to the viral load of its neighbors. Each individual has a coefficient of connectedness to determine the weight of the connection with each of its neighbors.

In this paper, we have adopted the Vickers −Osgood approach to construct a contagion model that includes immune response quality which is based on the equations developed by Wordarz. Below, we have identified specific parameter values that demonstrate how Wodarz’s model could be used to represent the immune response of individuals in a diverse population. We are using the Lévy and normal distributions to represent the quality of the immunocompetence of different age groups, thereby modeling the concept of Population Immune Response (PIR).

## Methods

### Population immune response

Population Immune Response (PIR) is a new concept that captures the collective immune response (IR) of individuals *p*_*i*_ in a population P represented by the superposition of individual immune responses. PIR is formally defined in Definition 1.1 below.

#### **Definition1.1**.

PIR $= \bigcup _{i=1}^{|P|} {IR}_{i}(t)\ \forall \ p_{i} \in P$ in which ${IR}_{i}=\{\dot {X_{i}}, \dot {Y_{i}}, \dot {V_{i}}, \dot {W_{i}}, \dot {Z_{i}} \}$ in which $\dot {X_{i}}, \dot {Y_{i}}, \dot {V_{i}}, \dot {W_{i}}$ and $\dot {Z_{i}}$ are functions from the set of Eqs. 1–5.

**Fig. 3:**
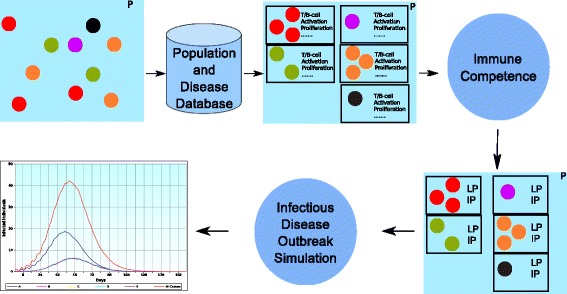
Outline of a computational model that applies the concept of PIR

Figure 3 summarizes how the effects of PIR on outbreak dynamics can be exploited and how the concept can be applied to create a computational model. The model is divided into three modules: *Population and Disease Database*, *Immune Competence*, and *Infectious Disease Outbreak Simulation*. A *Population and Disease Database* is required to store the population and disease information. This database must include demographic data for P that can be linked to the efficacy of an individual’s immune response and, hence, determine the collective immune responses of P. The database information is exploited to categorize the individuals into multiple clusters. These clusters are based on the demographic characteristics of interest in the study (eg. age, gender). Additionally, the database contains information related to the disease itself, such as pathogenicity and pathogen growth rate. The disease information is used to estimate the value of the transmission threshold *vbl*^∗^.

Once the clusters have been created, the immune response upon infection for each group is determined by the *Immune Competence* module. In this research, we exemplify this calculation with a mathematical approach to model the immune response of an individual during a viral infection. The model presented was first introduced by Arnaout et al. [30] and expanded by Wodarz [31]. This model captures the development of the disease in the host by portraying the interaction between host cells, viruses, and immune response. The interaction between *vbl* and the immune response is captured at a cellular level during the simulation of each cluster’s immune response. The result of every simulation will return the values of *vbl* for each cluster. From every *vbl* value it is possible to obtain the length of the disease periods for each cluster by incorporating *vbl*^∗^ as described in the previous section. Ultimately, the length of the disease period of each individual is calculated and incorporated into the simulation of the epidemic.

The *Infectious Disease Outbreak Simulation* will simulate the spread of a disease in a population during time *t*. The simulation must incorporate every individual from P and simulate the interactions between them. Each individual needs to be assigned unique characteristics representative of his or her individuality and social activities. Some of these characteristics are the length of the disease periods and the number of social interactions per time interval. These values are usually calculated in a per day share. The length of the disease periods of every individual will be assigned according to his or her cluster membership. The incorporation of different clusters in the simulations will result in different disease dynamics. Since the variation of length of the disease periods between individuals is determined by their immune response, we conjecture that PIR is a crucial driving force of the dynamics of an epidemic.

### Modeling the immune response

The module *Immune Competence* determines the infectious and latent period of individuals of every group from the population. To estimate those values it is possible to use models that capture the interaction between a pathogen and the immune response on a host. Multiple models have been proposed to simulate the immune system of an individual during infection [32]. In each model, different sets of parameters and interacting components are utilized to represent particular functions of the immune system.

#### Immune response model

To illustrate the concept of PIR, we sought a mathematical model sufficiently complex to capture the essential components of the immune response and capably represent different diseases. Arnaout et al. [30] introduced a mathematical model that separates the immune response into two different types: lytic and non-lytic response. A lytic immune response kills infected cells, whereas a nonlytic response prevents viral replication through soluble mediators [33]. The flexibility of this model allows the study of the effects of each response type during different disease infections. This mathematical model depicts the interaction between infected cells (*Y*), viruses (*V*), and the immune response. The immune response is represented as the quantity of virus-specific Cytotoxic T lymphocyte (CTL)(*Z*) and the virus-specific antibodies (*W*). Wodarz [31] expanded the model of Arnaout et al. by incorporating uninfected cells (*X*). The Wodarz model is represented by the set of differential Eqs. 1, 2, 3, 4 and 5. A description of the parameters of the model is presented in Table 1.

(1) $$\begin{array}{@{}rcl@{}}  \dot{X} & = & \lambda - d X - \beta X V \end{array} $$

**Table 1:** Description of the parameters from the Wordarz’s Model

Symbol	Definition
*λ*	Production rate of uninfected cells
*d*	Death rate of uninfected cells
*β*	Infection rate of uninfected cells by viruses
*a*	Death rate of infected cells
*p*	Lysis rate of infected cells by the CTL response
*k*	Production rate of virus by infected cells
*u*	Decay rate of viruses
*q*	Neutralization rate of viruses by antibodies
*g*	Development rate of antibodies in response of virus exposure
*h*	Decay rate of antibodies
*c*	Development rate of CTL in response to infected cells
*b*	Decay rate of CTL

(2) $$\begin{array}{@{}rcl@{}} \dot Y & = & \beta X V - aY - p Y Z  \end{array} $$

(3) $$\begin{array}{@{}rcl@{}} \dot V & = & k Y - u V - q V W  \end{array} $$

(4) $$\begin{array}{@{}rcl@{}} \dot W & = & g V W - h W  \end{array} $$

(5) $$\begin{array}{@{}rcl@{}} \dot Z & = & c Y Z - b Z  \end{array} $$

**Fig. 4:**
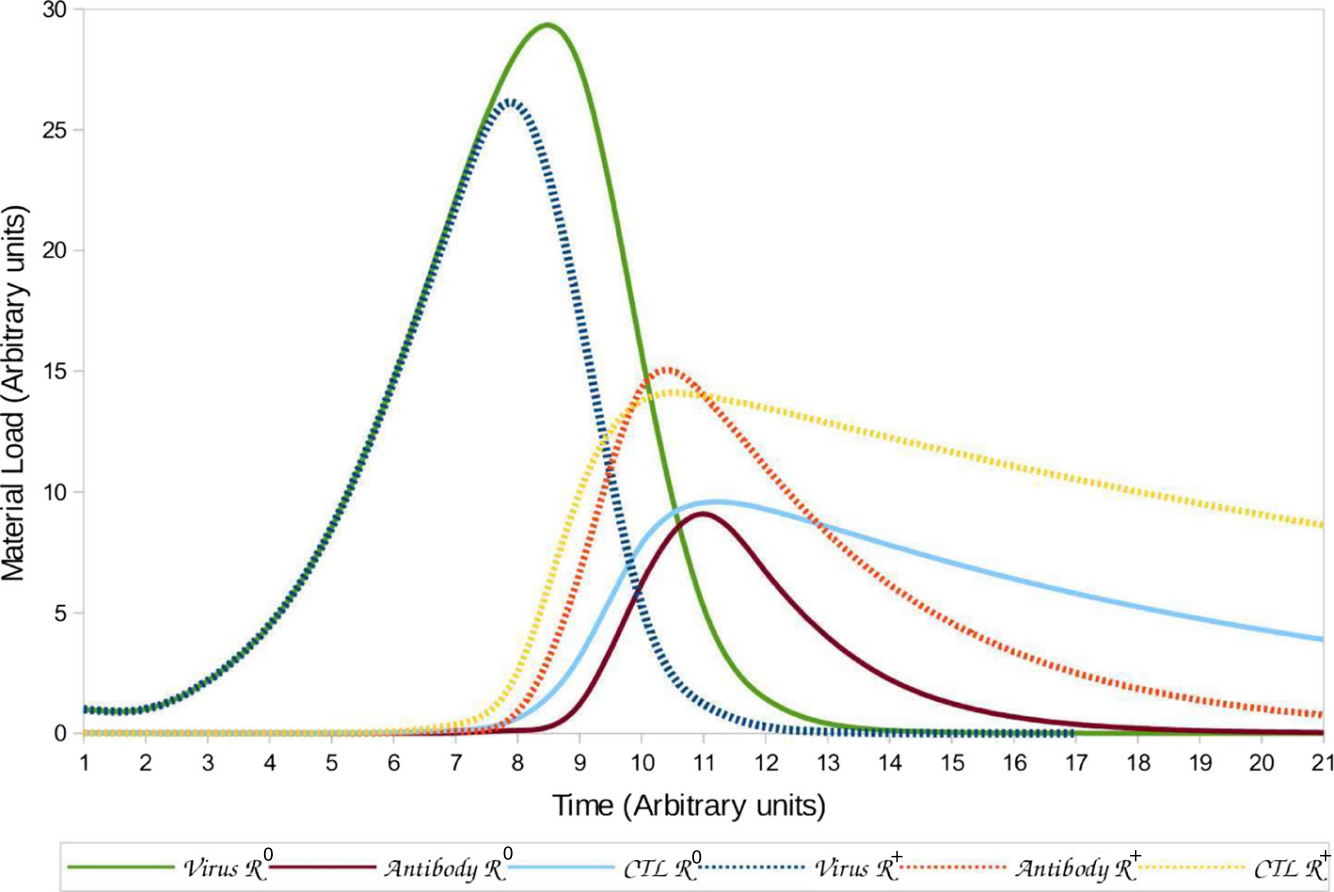
Resulting viral loads and quantities of antibodies and CTL by simulating the Wodarz model with parameters that represent a hyper immune response and a standard immune response

To study the immune system efficacy, we will focus on those parameters that determine the strength of the immune response. As described in the previous section, alterations of the immune system functionality are caused by multiple factors. For example, it is known that an individual will experience chronic involution of the thymus gland as he/she ages [34]. The involution of the thymus is considered one of the major reasons for the decline of immune response quality since the thymus is responsible for the production of naïve T-cells. Additionally, B-cell proliferation and efficacy are diminished due to the immunosenescence derived from aging [34]. Studies have shown a decrease of the B-cell population, reduction of antibody diversity, and decline of capacity to produce pathogen-specific antibodies as the individual ages [35, 36]. In the mathematical model, we represent this effect by incorporating individual age groups with different values for the immunological parameters. The immunological parameters of the model are: *g*, *q*, *b*, *h*, *q*, *c*, and *p*, as depicted in Table 1. Figure 4 depicts two simulation scenarios of the mathematical model for different values of the immunological variables. The simulation represents a standard immune response  and a hyperimmune response  The figure illustrates the performance of each response during infection and its impact on the viral load. Although there is evidence that the quality of those variables is affected by the age of the individual, determining its value only from age groups is not completely accurate. Multiple factors besides immunosenescence can be involved in establishing the strength of a specific immune response parameter; however, this is beyond the scope of this paper.

#### Modeling the immune response of the population

**Fig. 5:**
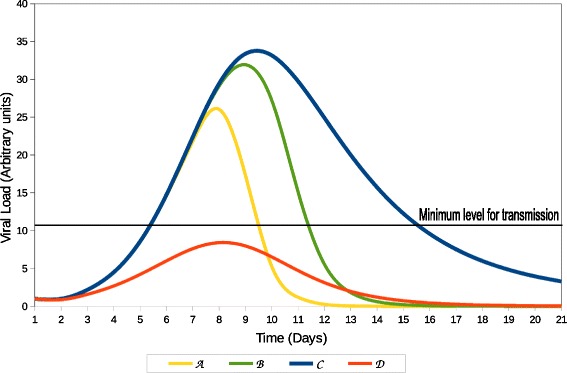
Viral load quantities for different types of qualities of the immune response obtained by simulating the Wodarz’s model and using the parameters presented in Table 2

As described above, the quality of the immune response of individuals is determined by multiple factors. However, given the scope of this research, we present an implementation that only considers the effects caused by immunosenescence with the goal to associate immunocompetence with demographic characteristics. This factor was selected given its importance is highlighted in multiple studies [34–37]. Additionally, data for the length of the infectious periods of multiple pathogens is commonly reported for different age groups [38, 39]. In this implementation, the quality of the immune response of individuals from different age groups was determined based on the reported data of disease periods for influenza. The World Health Organization (WHO) [40] reports that an infected adult (group *A*) is capable of transmitting the flu virus from 5 to 7 days. Similarly, infected children (group *B*) may infect others for up to 21 days with a median of 7–8 days and immunocompromissed individuals (group *C*) could be infectious for weeks or months. The immunocompromissed group includes adults 55 and older [41]. We included an additional group (group *D*) that represents individuals that have gained immunity to the disease either by natural or artificial immunization independent of their age. Considering that the infectious periods of each age group are determined based only on age, it is clear that the variability observed in the length of the infectious periods within the groups can be attributed to other factors such as gender, race, etc. Ultimately, each of the age groups is appointed to represent one type of immune response from the 4 types of immune response defined above, such that  and  Above, we exemplified the use of the Wodarz’s model as a method to determine the length of the infectious and latent periods of an individual. In this model, its parameters can be modified to simulate different immune responses with distinct qualities. For the disease described below, each age group is associated with multiple levels of immune response efficacies and efficiencies. Further, as depicted in the previously, the quality of the immune response can be represented by parameters *g*, *q*, *b*, *h*, *q*, *c*, and *p* from the mathematical model. Considering that the length of the infectious period varies for each age group, the parameters used to obtain the quality of the immune response of a group are not unique. Thus, the values for the parameters to simulate the immune response of an age group are calculated from a particular distribution of values. In Table 2 we present some of the possible values used to simulate the immune response of individuals from each group. However, the values for the simulations presented do not include all possible infectious periods lengths nor all possible values for the parameter from their distribution. Figure 5 depicts the resulting viral load values for the simulations with the parameter values presented in Table 2. Further, from Fig. 5 we obtain the latent and infectious period for each group by evaluating its intersection points with *vbl*^∗^. Assuming the time intervals as days, the latent period (*LP*) is 5 days for all groups. Equivalently, the length of the infectious period (*IP*) for each group is: *IP*_*A*_=5, *IP*_*B*_=7, *IP*_*C*_=11, and *IP*_*D*_=0. Members of group D can be infected; however, considering the prime quality of their immune response, they will never develop the disease and thus never become contagious. A similar process is necessary to obtain all other infectious periods for each age group.

**Table 2:** Values used in the model to simulate each immune response group

Symbol	A	B	C	D
*λ*	30	30	30	30
*d*	0.1	0.1	0.1	0.1
*β*	0.01	0.01	0.01	0.01
*a*	0.5	0.5	0.5	0.5
*p*	0.2	0.1	0.05	0.1
*k*	0.4	0.4	0.4	0.4
*u*	2	2	2	2
*q*	0.006	0.01	0.0025	0.005
*g*	0.1	0.09	0.025	0.5
*h*	0.3	0.6	0.3	0.3
*c*	0.015	0.01	0.003	0.006
*b*	0.05	0.1	0.02	0.05

**Fig. 6:**
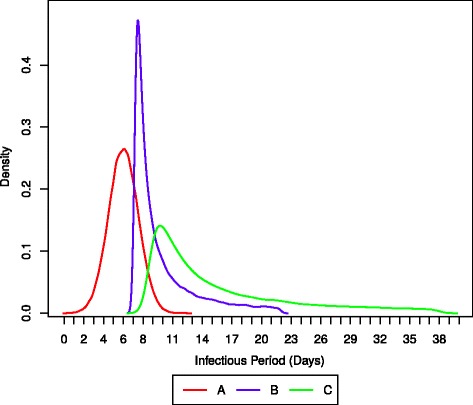
Probability distributions of the infectious period for the age groups A (14–55 years old), B (> 55 years old) and C (< 14 years old)

Determining the exact distributions for the parameters of the model in order to reflect the biological, sociological, and immunological characteristics of an individual is beyond the scope of this research. In this implementation, the lengths of the infectious period of each age group are calculated using probability distributions such that each distribution is an approximation to the data reported in the literature. Figure 6 depicts the probability distributions of the infectious period in each of the groups previously defined. Group A includes individuals between 14 and 55 years old and their infectious period is calculated with a normal distribution with mean *μ*=6 and standard deviation *σ*=1.5. Group B includes individuals with ages 0 to 14 and its represented with a Lévy distribution with location parameter *μ*=7 and scale parameter *c*=1. The Lévy distribution is depicted in Eq. 6. Finally, group C includes individuals 55 years old or more and their infectious period is calculated with a Lévy distribution with *μ*=8 and *c*=4. Ultimately, in the simulation every individual from each age group is assigned an infectious period following the probability distribution of its group.

(6) $$ f(x;\mu;c) = \sqrt{\frac{c}{2\pi}} \frac{e^{-\frac{c}{2(x-\mu)}}}{(x-\mu)^{\frac{3}{2}}}  $$

In the following section, we present multiple experiments to analyze some of the effects of PIR on the disease dynamics.

## Results and discussion

A series of experiments were conducted to exemplify the computational model proposed in the previous section. The simulation commences with the creation of a synthetic population. From this population, individuals are divided uniformly into four groups. Membership to three of the four groups is determined by the age of the individual. The three possible age groups are adult (*A*), children (*B*), and elderly (*C*). Affiliation to the fourth group is independent from the age of the individual but related to its immune status (*D*). All non-immune individuals are members of one of the other three groups (*A*, *B*, or *C*). Affiliation to a specific group was used to determine the quality of its immune response. Hence, the quality of the immune response of  and  as described above. We assume a disease in which the quality of the immune response of members from group *A* is more effective than those from group *B* and *C*. In the following experiments, the type of immune response and, consequently, the infectious period for each group is determined by the Lévy distribution and normal distribution as described above.

Once the lengths of the periods for each group have been determined, a computation of the spread of a disease within the groups is required. For this simulation, we utilized the Global Stochastic Contact Model (GSCM). The GSCM is a computational model that simulates the spread of an infectious disease in a population during an infectious outbreak [2]. The GSCM simulates the interactions between individuals in the population as the infection progresses. Based on multiple disease parameters, some of those interactions result in the transmission of the disease from individual to individual. In the model, multiple groups of individuals can be created. Each group is assigned values of specific disease parameters to represent heterogeneous populations. Some of the disease parameters include contact rate, transmissibility, affinity between clusters, and infectious and latent period lengths. For this simulation, we uniformly distributed the total population of 4000 individuals into the four groups previously described. Individuals from each group were assigned a respective latent and infectious period. Since this simulation is designed to explore the effects of the immune response, we assume all the other disease parameters to be identical for all groups.

### Experiment I

The first experiment explores the importance of incorporating different immune responses into the population. This experiment is divided into four cases, with each case consisting of a simulation of infectious outbreaks among homogeneous populations. The populations in each case consist of communities in which all individuals are members of only one of the immune response categories  In each case, individuals are assigned an infectious and latent period based on their immune response classification. All the other disease parameters are identical among all individuals for all cases. Each simulation started with the inclusion of a single infectious individual into the population. The result of every case is the average of 50 simulations. A run is considered an outbreak if more than 1 *%* of the population is infected. Otherwise, herd immunity or a deficient pathogen transmission is assumed. The results are summarized in Table 3. The table depicts the average number of infected individuals, the average number of individuals infected at the peak of the outbreak, the day in which the average peak occurred, and the average day in which the outbreak ended. The table displays an increased number of infected individuals as the quality of the immune response decrease. Additionally, the outbreaks with immunocompromised populations have an earlier peak and are shorter in duration compared to the other outbreaks, due to the heightened count of infected individuals in those experiments. In general, these results display the existence of a variation in the outbreak dynamics by incorporating an immune response to individuals in a population.

**Table 3:** Experiment 1 results: outbreak dynamics in homogeneous distributions of the population

(, Cluster)	Avg. Total Inf.	Avg. Inf. at Peak	Day of Peak	Avg. End of Outbreak
(, *A*)	637.78	17.47	210	563.84
(, *B*)	2979.29	384.24	128	301.16
(, *C*)	3579.45	844.84	117	258.22
(, *D*)	1	1	1	1

### Experiment II

**Fig. 7:**
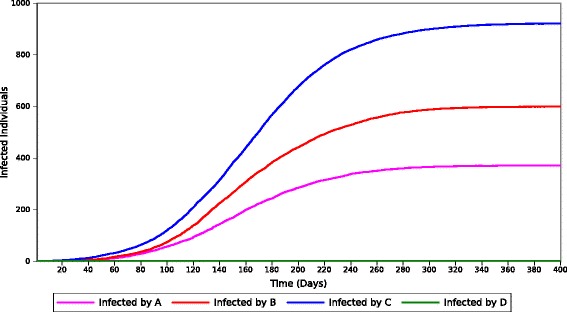
Cumulative number of individuals *infected by* members of every cluster of the population per day

The second experiment integrated groups *A*, *B*, *C*, and *D* to explore their effect on the disease dynamics. Since we are interested in measuring the effects of PIR in the disease dynamics, the rest of the characteristics of the population follow a homogeneous profile. To measure the effect of PIR, we computed the total number of infected individuals in each group and the proportion of the population that they infected. The results reported are the average over 50 simulations with different random seeds. As before, each simulation is initialized with a single infectious individual that is randomly assigned to one of the four groups. The results are summarized in Fig. 7 and Table 4. In the table, we observe that the distribution of the infected individuals is almost uniform. In contrast, the distribution of the individuals *infected by* members of each cluster is biased towards members from group *C*.

**Table 4:** Experiment 2 results: number of infected and *infected by* members of the four age groups in a heterogeneous mixing of the population

( Cluster)	Infected members	*Infected by* members of this cluster
(, *A*)	472.82	371.13
(, *B*)	472.86	599.68
(, *C*)	475.44	920.93
(, *D*)	471.62	0

In Fig. 7, we observe that members from group *C* are responsible for infecting the majority of individuals in the population. Individuals from this cluster also have the highest total infection rate at all times during the outbreak. These effects are a consequence of the incompetent immune response of the members of this group. This deteriorated response cannot control the viral growth, resulting in a larger than average *vbl* among its infected individuals. Due to the excessive quantity of pathogens, the level of *vbl* exceeds *vbl*^∗^ for a longer period of time as compared to the other two groups of individuals. Consequently, members from this group have an increased opportunity to infect others due their longer infectious period. Although the number of infected individuals in group *D* is similar to that of the other groups, members from this group present a special behavior. Since members from group *D* cannot transmit the infection to others, the number of individuals *infected by* members of this group is zero. Equation 7 depicts the relationship between infection rates among all clusters. Let  be the number of individuals that have been infected throughout the epidemic by groups *A*, *B*, *C*, *D* respectively. 
(7)

### Experiment III

**Fig. 8:**
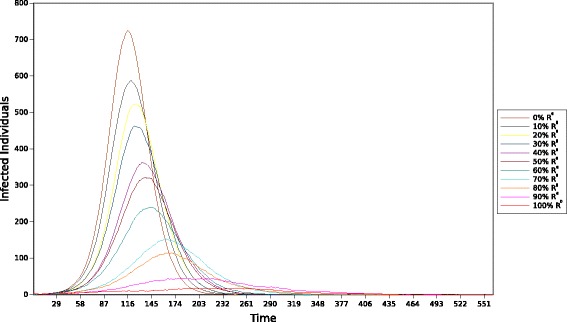
Resulting outbreak curves for different combinations of population distributions

The third experiment measures the role of PIR in a non-homogeneous population during an infectious outbreak. In this experiment, we represent different demographic distributions by splitting the population in three groups: *A*, *B*, and *C*. Group *D* is not included in this experiment since the role of this group is analyzed in Experiment IV. In Experiment III, we conducted 50 simulations in varied distributions of the the general population. The distributions are constructed by varying the number of individuals assigned to each group. The experiment commences by assigning all 4000 individuals from the population into group *A*. Consequentially, individuals will be removed from *A* and added to the other immune response groups (*B* and *C*) in increments of 5 *%* per each group until |*A*|=0. We present the results of these simulations, from an average of 50 runs with different seeds for each simulation, in Fig. 8 and Table 5.

**Table 5:** Experiment 3 results: outbreak dynamics during multiple simulations with different combinations of *A*, *B* and *C*

Population percentage			Population infected	Peak	Duration
(, *A*)	(, *B*)	(, *C*)	Total (Percentage)	Day (Infected Ind.)	Day
0	50	50	3547.42 (88.68)	117 (724.04)	213
10	45	45	3395.28 (84.90)	121 (587.42)	225
20	40	40	3289.58 (82.26)	127 (523.55)	234
30	35	35	3243.12 (81.09)	127 (461.9)	234
40	30	30	2962.73 (74.08)	134 (363.02)	254
50	25	25	2871.67 (71.80)	138 (321.45)	253
60	20	20	2578.54 (64.47)	145 (239.25)	271
70	15	15	2046.03 (51.16)	164 (152.75)	303
80	10	10	1766.96 (44.18)	170 (114.85)	317
90	5	5	1216.59 (30.42)	220 (44.36)	387
100	0	0	637.78 (15.94)	255 (17.42)	355

**Fig. 9:**
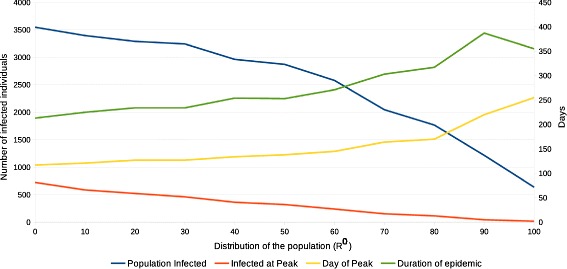
Resulting outbreak dynamics for different distributions of the population

Figure 8 depicts the number of infected individuals during multiple outbreaks with different combinations of *A*, *B*, and *C*. Figure 9 portrays the outbreak dynamics for the same distributions of the population. In both figures, we observe an increase in the size of the epidemic as more individuals from *A* are moved to *B* and *C*. Individuals from those two groups present a higher infectious period resulting in increased probabilities of infecting other individuals from the population. The figures also depict a variation in the duration and peak of the outbreak based on the number of individuals from *B* and *C*. The duration of the outbreak is reduced as the number of individuals in those groups increases. This effect is produced since individuals from those groups infect the susceptible individuals at a faster rate. This results in an exhaustion of susceptible individuals earlier in the outbreak.

Table 5 displays the total number and the percentage of infected individuals at the end of the epidemic, the day and number of infected individuals at the peak, and the duration of the outbreak. In the table, we observe that the proportion of infected individuals increases as a larger number of individuals from *B* and *C* are introduced to the population. The results indicate that the presence of more individuals with a weakened immune response in the population not only affect the duration and magnitude of the outbreak, but the percentage of infections as well.

### Experiment IV

**Fig. 10:**
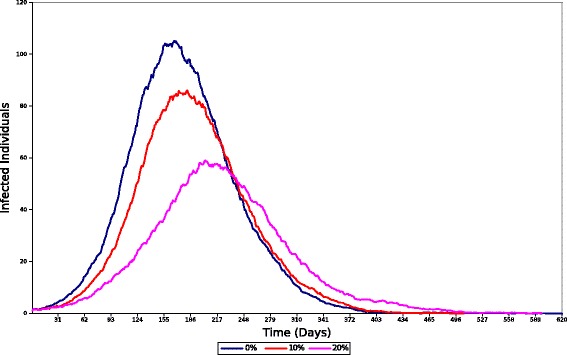
Total number of infected individuals from the population per day after different vaccination strategies

**Fig. 11:**
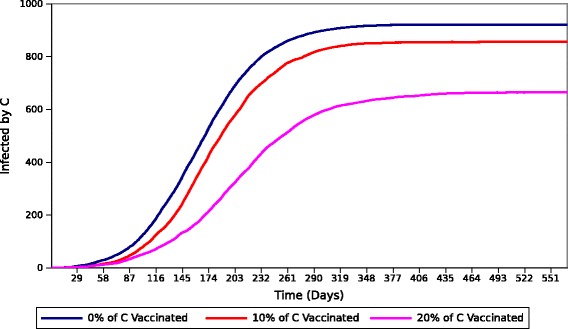
Cumulative number of individuals *infected by* members from group C after different vaccination strategies

The fourth experiment attempts to measure the effect individuals from group *C* have on the outbreak at large under the assumption of different vaccination strategies. Targeted vaccination of individuals from group *C* was selected since the previous experiments showed that members from this group cause the majority of the infections in the population; thus, generating interest in containing the force of infection originated from individuals in this group. Three vaccination strategies were implemented to represent different intervention scenarios. Each strategy consists of removing a percentage of individuals from group C and incorporating them into the immunized group (*D*). In this paper is assumed that the population was all vaccinated before the onset of the epidemic. Each vaccination strategy removed 0 *%*, 10 *%* and 20 *%* of the members from *C*, respectively. The 0 *%* strategy represents a zero-intervention scenario. This scenario is simulated utilizing the same population and values of disease parameters as in Experiment II. The other two intervention strategies also use the same values of disease parameters for each group, but with a modified population distribution. Similar to the previous experiments, a single infectious individual was randomly assigned to a group at the beginning of each simulation. The final result was obtained by averaging the outbreak dynamics over 300 runs for every strategy. The results from this experiment are divided in two parts: total number of infected individuals in P and total number of individuals *infected byC*. The results are depicted in Figs. 10 and 11, respectively.

Figure 10 illustrates the different outbreaks in P after each vaccination strategy is implemented. We observe that the 0 *%* strategy produces the greatest number of infected individuals and results in the highest infection rate per day compared to the other strategies. On the other hand, the 20 *%* strategy is more effective at limiting the progression of the disease due to its high number of members in group *D*. This results in the lowest number of infected individuals among all strategies. Further, we observe a variation in the length of the outbreak for each strategy. The 0 *%* strategy results in the shortest outbreak compared to the other two strategies. This effect is caused by the increased number of individuals with weak immune response in that strategy. The further reduction of individuals from *C*, in the other two strategies, results in a reduced opportunity to infect susceptible individuals that could, potentially, spread the disease even further. Consequently, the number of infections is reduced and the pathogen spread less aggressively, providing a larger population of susceptible individuals for future transmissions as opposed to populations with a more aggressive progression.

Figure 11 illustrates the cumulative number of individuals *infected by* members from group *C* for each vaccination strategy. Here, the number of individuals *infected by* group *C* behaves similarly to the total number of infected individuals in Fig. 10. We observe that the 0 *%* strategy causes the greatest number of individuals *infected by* members from group *C*. Furthermore, the 10 *%* and 20 *%* strategies decrease that count according to the intervention type, resulting in a reduced number of individuals *infected by* group *C*. More importantly, this result confirms the strong relationship between this group and the general outbreak. We observe that Figs. 10 and 11 display similar patterns for each respective outbreak. In general, these results indicate that if only a limited number of vaccines are available or vaccination resources are limited, the vaccination should be targeted towards the individuals that are most likely to spread the disease over longer periods of time. More realistic strategies, such as gradual roll-out vaccination, can be applied to study the effect of high-risk groups to the outbreak at large, but this is beyond the scope of this research and will be explored elsewhere.

## Conclusion

In this paper, we highlight the importance of integrating the viral/bacterial load into an infectious outbreak simulation. This value is utilized to measure the progression of the disease in the host and the host’s capacity of transmitting it. More importantly, we emphasized the direct relationship between the quality of the immune response and the quantity of the viral/bacterial load. Our results indicate that stronger immune response controls the growth of the pathogen more efficaciously and therefore affects the length of the infectious periods. For the experiments described in this paper, we assume a transmission threshold for the viral/bacterial load, above which an individual is capable of shedding the pathogen. Once the viral/bacterial load surpasses this threshold, the individual will become infectious and able to infect others until his or her load falls below that threshold. Hence, individuals with a hypo-immune response function can be assumed to propagate the disease for a longer period of time.

In this paper we introduced the new concept of Population Immune Response (*PIR*). PIR captures the collective immune response of individuals in a population represented by the superposition of individual immune responses. A computational model that captures the effects of PIR on the outbreak dynamics has been presented. The model is divided into 3 compartments: *Population and Disease Database*, *Immune Response Model*, and *Infectious Disease Outbreak Simulation*. Ultimately, the output of this model is the simulation of the disease dynamics during the infectious outbreak.

Multiple experiments were conducted to analyze some of the effects PIR has on the disease dynamics. The first experiment explores the variation in the outbreak dynamics as a consequence of incorporating different types of immune response into all individuals in a homogeneous population. The next set of experiments divided the population into three age groups and an immunized group. Each group was characterized by a unique immune response quality and thus a different length for its immune periods. A simulation experiment was conducted to study the spread of a disease within and among each of the groups. The experiments showed that individuals with weak immune responses and those who are immune to the pathogen play a significant role in shaping the outbreak dynamics. Finally, we explored the effects of incorporating targeted vaccination into the model by implementing different vaccination strategies directed to the individuals that are most likely to spread the disease over longer periods of time. The results suggest that if vaccination resources are limited, the vaccination should be targeted towards those individuals.

In general, our results suggest that it is essential for the public health establishment to increase their understanding of the characteristics of regional demographics, specially those that could impact the quality of the immune response of the individuals. The results indicate that it is necessary to further investigate mitigation strategies to limit the capacity to transmit the disease by individuals that are more likely to spread the pathogen for extended periods of time since they play a key role in the epidemic at large. Ultimately, this study suggests that it is imperative for public health researchers to identify appropriate targeted vaccination regimes and to explore the link between PIR and outbreak dynamics to improve the monitoring and mitigating efforts of ongoing and future epidemics.

## Abbreviations

average immune response
hyperimmune response
hypoimmune response
immune
*vbl*: viral/bacterial load
*v**b**l*^∗^: transmission threshold
PIR: population immune response
*LP*: latent period
*IP*: infectious period
*IP*_*A*_: infectious period of group A
■■■: Model parameters are listed in Table 1
